# Novel prognostic biomarkers in nasopharyngeal carcinoma unveiled by mega-data bioinformatics analysis

**DOI:** 10.3389/fonc.2024.1354940

**Published:** 2024-05-24

**Authors:** Yishuai Tan, Jiao Zhou, Kai Liu, Ruowu Liu, Jing Zhou, Zhenru Wu, Linke Li, Jiaqi Zeng, Xuxian Feng, Biao Dong, Jintao Du

**Affiliations:** ^1^ Department of Otolaryngology-Head & Neck Surgery, West China Hospital, Sichuan University, Chengdu, China; ^2^ West China School of Pharmacy, Sichuan University, Chengdu, China; ^3^ Department of Geriatrics, West China Hospital, Sichuan University, Chengdu, China; ^4^ College of Biomedical Engineering, Sichuan University, Chengdu, China; ^5^ Institute of Clinical Pathology, Key Laboratory of Transplant Engineering and Immunology, NHC, West China Hospital, Sichuan University, Chengdu, Sichuan, China; ^6^ State Key Laboratory of Biotherapy, West China Hospital, Sichuan University, Chengdu, China

**Keywords:** nasopharyngeal carcinoma, survival model, prognosis, mega-data bioinformatic analysis, scRNA-seq

## Abstract

Nasopharyngeal carcinoma (NPC) is commonly diagnosed at an advanced stage with a high incidence rate in Southeast Asia and Southeast China. However, the limited availability of NPC patient survival data in public databases has resulted in less rigorous studies examining the prediction of NPC survival through construction of Kaplan-Meier curves. These studies have primarily relied on small samples of NPC patients with progression-free survival (PFS) information or data from head and neck squamous cell carcinoma (HNSCC) studies almost without NPC patients. Thus, we coanalyzed RNA expression profiles in eleven datasets (46 normal (control) vs 160 tumor (NPC)) downloaded from the Gene Expression Omnibus (GEO) database and survival data provided by Jun Ma from Sun Yat-sen University. Then, differential analysis, gene ontology (GO) enrichment, Kyoto Encyclopedia of Genes and Genomes (KEGG) pathway analysis and network analysis were performed using STRING database. After that, 2142 upregulated differentially expressed genes (DEGs) and 3857 downregulated DEGs were screened. Twenty-five of them were identified as hub genes, which were enriched in several pathways (cilium movement, extracellular matrix structural constituent, homologous recombination and cell cycle). Utilizing the comprehensive dataset we amassed from GEO database, we conducted a survival analysis of DEGs and subsequently constructed survival models. Seven DEGs (RASGRP2, MOCOS, TTC9, ARHGAP4, DPM3, CD37, and CD72) were identified and closely related to the survival prognosis of NPC. Finally, qRT-PCR, WB and IHC were performed to confirm the elevated expression of RASGRP2 and the decreased expression of TTC9, CD37, DPM3 and ARHGAP4, consistent with the DEG analysis. Conclusively, our findings provide insights into the novel prognostic biomarkers of NPC by mega-data bioinformatics analysis, which suggests that they may serve special targets in the treatment of NPC.

## Introduction

Nasopharyngeal carcinoma (NPC) is a distinct head and neck cancer, with patients mainly from East Asia, Southeast Asia, and North Africa (1). The incidence and mortality of NPC in China is high globally. An estimated 42,100 new cases and 21,320 deaths were attributed to NPC in China in 2013, accounting for 1.14% of all new cancer cases and 0.96% of all cancer-related deaths (2).

NPC is increasingly considered an ecological disease, with the tumor microenvironment (TME) playing a pivotal role in the cancer’s progression (3). The NPC microenvironment is characterized by an increased abundance of suppressive regulatory T cells (Tregs) (4). Furthermore, recent research indicates that malignant epithelial cells can activate Tregs through the CD70-CD27 pathway, exacerbating immune suppression (5). Innate-like B cell has been identified as a potential biomarker for gemcitabine-plus-platinum (GP)-based treatment in NPC, which could improve patient management (6). Professor Ma Jun’s team has made further refinements to the 8th edition of the AJCC/UICC TNM classification, which holds promise for enhancing patient treatment outcomes (7). Moreover, due to the lack of clinical information specific to NPC, most of studies have utilized data from head and neck squamous cell carcinoma (HNSCC) for survival analysis. However, this approach is flawed as the HNSCC database contains minimal NPC data, a tumor type distinct from HNSCC, rendering the survival results largely irrelevant (8–11). NPC can be effectively treated in early stages, yet over 50% of patients are diagnosed at advanced stages, leading to worse outcomes and prognosis (12). Consequently, there is a critical need for the identification of biomarkers that enable early diagnosis and prognostication, thus guiding high-risk individuals towards timely follow-up and intervention.

Recently, microarrays and high-throughput sequencing have become powerful and effective tools that allow the detection of genome-wide gene expression differences in healthy populations and cancer patients (13). It is possible to define the gene expression profile of the tumor and to correlate it with prognosis and metastasis (14). The Gene Expression Omnibus (GEO) repository at the National Center for Biotechnology Information (NCBI) archives and freely distributes high-throughput molecular abundance data, predominantly gene expression data generated by DNA microarray technology (15). The cBioPortal for Cancer Genomics (http://cbioportal.org) provides a Web resource for exploring, visualizing, and analyzing multidimensional cancer genomics data (16). However, there is a lack of multiple database combination analysis and screening of DEGs from mega data.

Hence, samples of NPC from the GEO database were collected and analyzed based on the principle of bioinformatics to screen the differentially expressed genes (DEGs). Combined with the GEO database and the clinical information with related NPC RNA profile data from Jun Ma (17), we used these screened genes to perform subsequent survival analysis. Multivariate Cox regression analysis was performed to verify the credibility of the DEGs. Finally, seven DEGs (RASGRP2, MOCOS, TTC9, ARHGAP4, DPM3, CD37, and CD72) were identified and closely related to the survival prognosis of NPC. Subsequently, the survival models constructed by the above identified DEGs predicted patient prognosis well. Therefore, our study constructed a prognostic model and identified novel prognostic biomarkers of NPC by mega-data bioinformatics analysis, which suggests that they may serve special targets in the treatment of NPC.

## Materials and methods

### Data acquisition and principal component analysis

Microarray datasets (GSE12452 (18), GSE64634 (19), GSE15047, GSE15903 (20), GSE36972 (21), GSE48501 (22), GSE100193 (23), GSE79571 (24), GSE119020 (25), GSE127848, GSE128502 (26), GSE13597 (27), GSE53819 (28), GSE40290, GSE34573 (29)) were accessed from the GEO database (http://www.ncbi.nlm.nih.gov/geo/), which is an international public repository for high-throughput microarray and next-generation sequence functional genomic datasets submitted by the research community (30). Because these datasets belong to different Geo platforms (GPL) and the number of genes varies greatly between platforms, we combined datasets that belong to the same platform (GPL570) into a group (MIX) to avoid the loss of a large number of genes. Finally, these datasets were divided into five groups for further experiments: GSE34573, GSE53819, GSE13597, GSE40290 and MIX (GSE34571: 3 normal samples and 14 tumor samples, GSE53819: 18 normal samples and 18 tumor samples, GSE13597: 3 normal samples and 25 tumor samples, GSE40290: 8 normal samples and 25 tumor samples, and MIX: 14 normal samples and 78 tumor samples). Because the MIX group’s data came from different datasets, we used limma packages to remove batch effects. Meanwhile, GSE102349 and GSE132112 were obtained for the survival analysis. More detailed sample information and grouping results are presented in **Table 1**. PCA was conducted via R package FactoMineR and factoextra, which was further visualized by using scatterplot3d function from R package scatterplot3d.

**Table 1:** Details of the five groups.

Dataset	Platform	PMID	Sample type	group
GSE12452	GPL570 [HG-U133_Plus_2] Affymetrix Human Genome U133 Plus 2.0 Array	17119049	NPC	MIX
GSE64634	GPL570 [HG-U133_Plus_2] Affymetrix Human Genome U133 Plus 2.0 Array	26246469	NPC	MIX
GSE15047	GPL570 [HG-U133_Plus_2] Affymetrix Human Genome U133 Plus 2.0 Array	unknown	CNE, HONE	MIX
GSE15903	GPL570 [HG-U133_Plus_2] Affymetrix Human Genome U133 Plus 2.0 Array	19963132	NPC	MIX
GSE36972	GPL570 [HG-U133_Plus_2] Affymetrix Human Genome U133 Plus 2.0 Array	23069661	NPC 5-8F	MIX
GSE48501	GPL570 [HG-U133_Plus_2] Affymetrix Human Genome U133 Plus 2.0 Array	24498188	NPC	MIX
GSE100193	GPL570 [HG-U133_Plus_2] Affymetrix Human Genome U133 Plus 2.0 Array	28906488	CNE	MIX
GSE79571	GPL570 [HG-U133_Plus_2] Affymetrix Human Genome U133 Plus 2.0 Array	28423621	HONE	MIX
GSE119020	GPL570 [HG-U133_Plus_2] Affymetrix Human Genome U133 Plus 2.0 Array	30683844	HONE	MIX
GSE127848	GPL570 [HG-U133_Plus_2] Affymetrix Human Genome U133 Plus 2.0 Array	unknown	NPC	MIX
GSE128502	GPL570 [HG-U133_Plus_2] Affymetrix Human Genome U133 Plus 2.0 Array	31321241	NPC-TW01	MIX
GSE13597	GPL96 [HG-U133A] Affymetrix Human Genome U133A Array	19142888	NPC	GSE13597
GSE53819	GPL6480 Agilent-014850 Whole Human Genome Microarray 4x44K G4112F	24763226	NPC	GSE53819
GSE40290	GPL8380 Capitalbio 22K Human oligo array version 1.0	unknown	NPC	GSE40290
GSE34573	GPL570 [HG-U133_Plus_2] Affymetrix Human Genome U133 Plus 2.0 Array	22815911	NPC	GSE34573

### Selection and identification of DEGs

We performed a differentially expressed gene (DEG) analysis of each of the five groups through the limma package with filter criteria: log2FC>1 and p value<0.05. The limma package is an R/Bioconductor software package that provides an integrated solution for analyzing data from gene expression experiments (31). Then, the ComplexHeatmap function was used to display the top 50 upregulated and top 50 downregulated DEGs of each group (rank according to log fold change). Meanwhile, we continued to show the genes that met the screening criteria through the EnhancedVolcano package. The Venn diagram presents the overlap of DEGs using jveen (http://jvenn.toulouse.inra.fr/) online tools.

### Enrichment analysis for nasopharyngeal carcinoma

To further acknowledge the significant role that DEGs play in biological functions, enrichment analysis was carried out. We adopted clusterProfiler together with GOplot to achieve Gene Ontology (GO) term enrichment analysis, which was divided into three types: biological processes (BP), cellular components (CC), and molecular functions (MF). Kyoto Encyclopedia of Genes and Genomes (KEGG) pathway analysis was also performed. Furthermore, we also show the significant DEGs in the key pathways through the function GOchord from the GOplot R package.

### Construction of the protein−protein interaction network and TF-gene network

Overlapping DEGs that appeared in at least four groups were used to construct the PPI network by STRING (STRING: functional protein association networks (string-db.org)). They are also displayed in **Table 2**. We prefer to achieve a comprehensive and objective global network, including direct (physical) as well as indirect (functional) interactions for DEGs by STRING (32). Cytoscape software was used to calculate the degree of connectivity between DEGs to identify hub genes. Concurrently, we built the TF-gene network of DEGs in NetworkAnalyst (www.networkanalyst.ca), which is a Web-based tool for creating and visualizing biological networks, and users can now perform gene expression profiling for 17 different species (33).

**Table 2:** Overlapping differentially expressed genes (DEGs).

Regulation	Overlapped differentially expressed genes(DEGs)
Up-regulated DEGs (appear in five groups^*^)	KIF23, TOP2A, BUB1B, RBBP8, PRC1, GALNT11, VRK2, NFE2L3, TNFAIP6, LAMB1
Up-regulated DEGs (appear in four groups^#^)	CDK1, ASPM, RAD51AP1, LMNB2, DTL, NPL, ZWINT, PMAIP1, FJX1, GINS1, STAR, ECT2, RCN1, CCL8, COL4A1, ZIC2, IDH1, GRB10, COL5A2, USP18, TTK, PLAU, MMP1, PTGS2, CCNB2, TPX2, TMPO, GJA1, BRCA1, INSM1, GAD1, KIF14, LHX2, TFRC, PUS7, MEST, FOXM1, ITGAV, ROBO1, KREMEN2, GADD45A, ARNT2, RAI14
Down-regulated DEGs (appear in five groups^*^)	MSLN, LTF, SCGB1A1, CRYM, SPAG6, CYP2F1, CYP4B1, AGR2, SLPI, MUC13, MSMB
Down-regulated DEGs (appear in four groups^#^)	UPK1B, ALOX15, KRT4, CDH26, CAPS, ALDH3A1, DHRS9, MUC20, RRAD, TEKT1, FAM3D, S100P, DNAI1, SPA17, ROPN1L, BCAS1, ZMYND10, CH25H, CHST9, CXCL14, IQCD, C9orf116, MAL, HSPB8, CEACAM6, GCNT3, C4orf19, DNALI1, SCGB2A1, ALDH1L1, PIP, TMC5, CLDN10, ATP2C2, FOLR1, TSPAN1, PACRG, MUC16, TFF3, SCGB3A1, ZMYND12, RIBC2, SLC22A4, AK7, FUT3, VILL, MLPH, SELENBP1, ANG, NDRG2, TPPP3, NME5, LCN2, SERPINB7, ADRA2A, PROM1, FOXJ1, BLK, CR2, ATP2A3, AQP5, CLU, PIGR

*including GSE34573, GSE53819, GSE13597, GSE40290 and MIX; # Four of GSE34573, GSE53819, GSE13597, GSE40290 and MIX.

### Manufacture of the DEG mutation spectrum diagram

To investigate the mutation standard of the overlapping DEGs, we constructed the mutation signature by cBioProtal online tools. The high mutation frequency in DEGs is shown based on NPC mutation datasets. This dataset was obtained from a study of the genomic landscape of NPC (34).

### Gene screening based on clinical information

Two datasets, GSE132112 (17) and GSE 13597, were chosen for further research on DEGs on account of clinical information. Different radiotherapy and chemotherapy strategies should be applied to patients with different stages (35). Accordingly, we studied the effects of DEG quantity on stage and metastasis to assist in choosing the method of treatment. Thus, we plotted a boxplot of genes associated with tumor-node-metastasis (TNM) staging using the ggstatsplot R package. The DEGs that were strongly associated with staging were further analyzed for survival.

### Survival prognostic analysis for DEGs

The Kaplan−Meier curve is a comparative analysis that depends upon the whole curve and not upon isolated points (36). The genes used to plot the curves were those identified by differential analysis. Meanwhile, some of the differentially expressed genes (DEGs) were related to the stages and grades of the patients or had a high hazard ratio. The progression-free survival information was downloaded from GSE102349 (37), which provides 113 treatment-naïve undifferentiated NPC tumor gene expression matrices and clinical information. The overall survival (OS) and failure-free survival were completed using GSE132112. All survival prognoses were analyzed for selected DEGs by the survival and survminer R packages.

### Analysis of high-risk genes based on crucial cancer clinical trial endpoints

Multivariate Cox regression analysis was constructed to forecast the patient’s prognosis. The above three models were arranged for overall survival (OS), progression-free survival (PFS), and failure-free survival (FFS). At the same time, patients were divided into low- and high-risk groups according to the median risk score (38, 39). The risk score was calculated as follows: risk score = exp(1)*h(1) + exp(2)*h(2) + exp(3)*h(3) + exp(4)*h(4) + … + exp(n)*h(n), where exp(x) represents the gene expression level, and h(x) represents the regression coefficient calculated by multivariate Cox proportional hazard regression). These key genes are integrated to build risk models.

### Single cell sequencing data analysis for nasopharyngeal carcinoma

The public sequencing data were downloaded from the Human Cell Atlas(4 nasopharynx) (40) and GEO (GSE162025 (41): 1 nasopharynx and 5 nasopharyngeal carcinoma). All scRNA-seq data meet the quality control criteria: chondriosome gene lower than 5% and the number of total genes is between 200 and 5000. We use R package Seurat (v 4.3.0) to merge the three scRNA seq data and analyze the spatial transcriptome data. Then, R package harmony (42) was used to remove the batch effects of the three different origin datasets. Expression levels visualization was using R package ggplot2, Seurat and scCustomize.

### NPC sample collection and isolation of total RNA for real-time quantitative PCR

Seven NPC samples and four control samples were collected. All patients signed informed consent forms, which were approved by the hospital ethics committee. The diagnosis of all patients was confirmed by pathology. Total RNA from specimens was isolated using the FastPure Cell/Tissue Total RNA Isolation Kit V2 (Vazyme, Nanjing, China) according to the manufacturer’s instructions. mRNA to cDNA was synthesized using Bimake All-in-One cDNA Synthesis SuperMix (Bimake, Houston, USA), and qPCR of mRNAs was performed with Bimake 2x SYBR Green qPCR master mix (Bimake). Relative expression was calculated with the 2−ΔΔCT method, and levels were normalized using GAPDH for mRNAs. The sequences of primers used in this study are shown in **Table 3**.

**Table 3:** PCR primers.

	Forward primer	Reverse primer
GABRP	TTTCTCAGGCCCAATTTTGGT	GCTGTCGGAGGTATATGGTGG
PTPN6	TGAACTGCTCCGATCCCACTA	CACGCACAAGAAACGTCCAG
ARHGAP4	GATGACGGGGATGTGCTTGAT	GGTAGAAGGTTTCGGTCTCCTG
TTC9	TGAAGCCATAGGCAAATACCAC	TGACTCGTTCATAGTTTACCAGC
CD37	TCCTGAGAGGTAACGGGTCG	GGATTGTGGAGTCGTTGGTCG
DTL	TGGTCTTCACAATACCCTCTTCA	CTTCATTGGCAACTGCTAGTACA
RASGRP2	TCGCCTGTCAGTTGAGTGTC	CCAGTAGCCCTGCATCCTTC
DPM3	GGCCACTTTTCATGACTGCG	GAAATGGGAGGAAGGGCTGT

### Western blotting

After being thoroughly grounded in liquid nitrogen, the tissues were collected for western blotting (WB). Total protein of tissues or cells were extracted in RIPA lysis buffer with a cocktail of protease inhibitors (Bimake), and then boiling for 10 min with SDS loading buffer. Equal amounts of protein were electrophoresed on SDS-PAGE in 10% Tris‐glycine gels and transferred to PVDF membranes (Millipore, MA, USA). Membranes were blocked with 5% non-fat milk in tris buffered solution containing 0.1% Tween-20 at room temperature for 1 h, followed by overnight incubation with primary antibodies against GAPDH, anti-KIF2C (Proteintech) antibody, anti-CD37 antibody (HUABIO), anti-SHP1 (PTPN6) antibody (HUABIO), CDT2 (DTL) rabbit mAb (ZENBIO), and ARHGAP4 rabbit pAb (ZENBIO). After washing thrice at room temperature, the membranes were incubated with secondary antibody (Zen Bioscience, Chengdu, China) and signals were visualized by using ECL Plus Western Blotting Reagent Pack (Bio-Red, Hercules, USA). The band intensities were quantified by Fusion Solo Imaging System (VIBER LOURMAT, FRANCE).

### Immunohistochemistry and statistical analysis

Immunohistochemistry (IHC) staining was carried out using the Biotin-Streptavidin horseradish peroxidase Detection Kit (SP-9001, ZSGB-Bio, Beijing, China) and the DAB Detection Kit (ZLI-9061, ZSGB-Bio). For each section, three fields of view were selected, and images were captured using a microscope at 200× magnification. The tissue sections were subjected to immunostaining using the following primary antibodies: anti-KIF2C polyclonal antibody (1:200, Proteintech), anti-CD37 antibody (1:100, HUABIO), anti-SHP1 (PTPN6) antibody (1:100, HUABIO), CDT2 (DTL) rabbit mAb (1:100, ZENBIO), and ARHGAP4 rabbit pAb (1:100, ZENBIO). Immunohistochemical staining intensity was quantified by calculating the average rate of positively stained cells using ImageJ and IHC Profiler across three distinct high-power fields (200× magnification) on each slide (**Supplementary Table S1**). The median immunoreactivity score for each gene served as the threshold for our analyses. We assessed prognostic variables through bivariate Kaplan-Meier log-rank tests and multivariate Cox proportional hazards modeling. Statistical significance was set at a two-sided P value of less than 0.05. Survival curves were conducted using GraphPad Prism version 9.

## Results

### Identification of differentially expressed genes between nasopharyngeal carcinoma and normal tissues after data standardization

**Figure 1:**
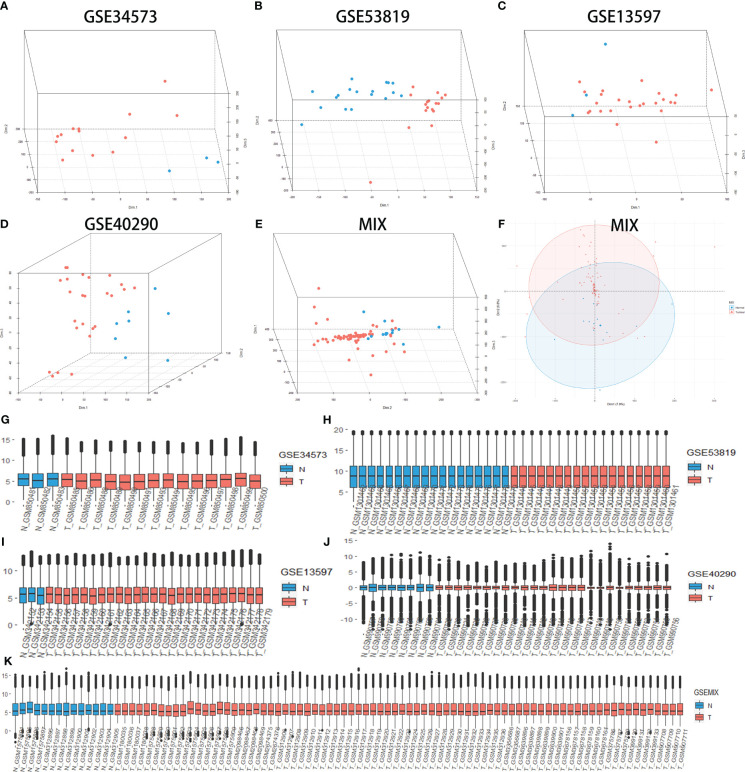
Principal component analysis (PCA) shows the clustering of different samples in the RNA expression matrix in different groups, quality control (QC) for all groups. PCA between nasopharyngeal carcinoma (NPC) and normal tissue samples (control) in GSE34573 **(A)**, GSE53819 **(B)**, GSE13597 **(C)**, GSE40290 **(D)** and MIX **(E, F)**. Blue plots represent normal tissue, and red plots represent NPC. QC for GSE34573, GSE53819, GSE13597, GSE40290 and MIX **(G-K)**. The red box represents normal tissue, and the cyan box represents NPC.

Principal component analysis (PCA) was performed to determine whether cancer and normal samples could be separated in each group of data. The results showed an apparent distinction between NPC and control tissue (**Figures 1A–F**). Then, the expression value of each group was designed as a boxplot with the sample median and expression value being similar in each group, which means that the quality of each group of data was acceptable (**Figures 1G–K**). Jointly, these results demonstrated that our grouping was reasonable and met the conditions for further identification of DEGs.

**Figure 2:**
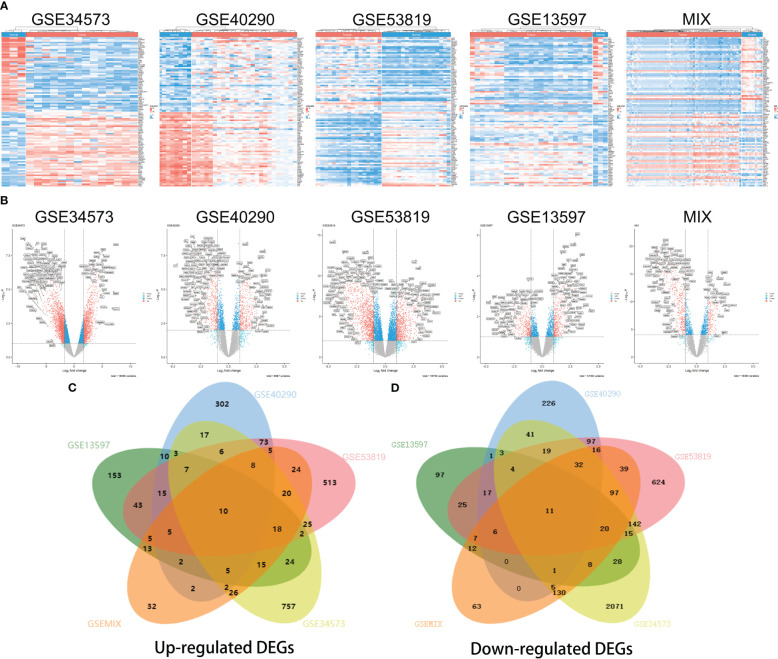
Heatmaps and volcano plots for screening differentially expressed genes (DEGs) in different datasets. Heatmap of GSE34573, GSE40290, GSE53819, GSE13597 and MIX **(A)**, which display the top 50 downregulated DEGs (on the top) and top 50 upregulated DEGs (on the bottom) for each group. Volcano plots of the distributions of DEGs in GSE34573, GSE40290, GSE53819, GSE13597 and MIX **(B)**. DEGs are mapped as red plots. Venn diagram of upregulated DEGs and downregulated DEGs among the five groups GSE34573, GSE40290, GSE53819, GSE13597 and MIX **(C, D)**.

Heatmaps of the top 50 upregulated DEGs and top 50 downregulated DEGs in each group are shown (**Figure 1A**). Additionally, the volcano plots showed that DEGs were differentially expressed between NPC and control tissue samples with the following filter criteria: |log2(fold change)| >1 and p.value<0.05 (**Figure 2B**). All DEGs from the five groups were plotted as Venn diagrams (**Figures 2C, D**). The overlapping DEGs that appeared in at least four groups and the other overlapping DEGs are listed in the supplement (**Supplementary Data Sheets 1**, **2**). Eventually, we identified 2142 upregulated genes and 3157 downregulated genes (filter criteria: log2(fold change) >1 and p.value<0.05). A more stringent filter of |log2(fold change)| > 1.5 with the same p-value criterion revealed 1,316 upregulated and 2,718 downregulated genes (**Supplementary Data Sheet 3**).

### Functional enrichment analysis of overlapping DEGs

**Figure 3:**
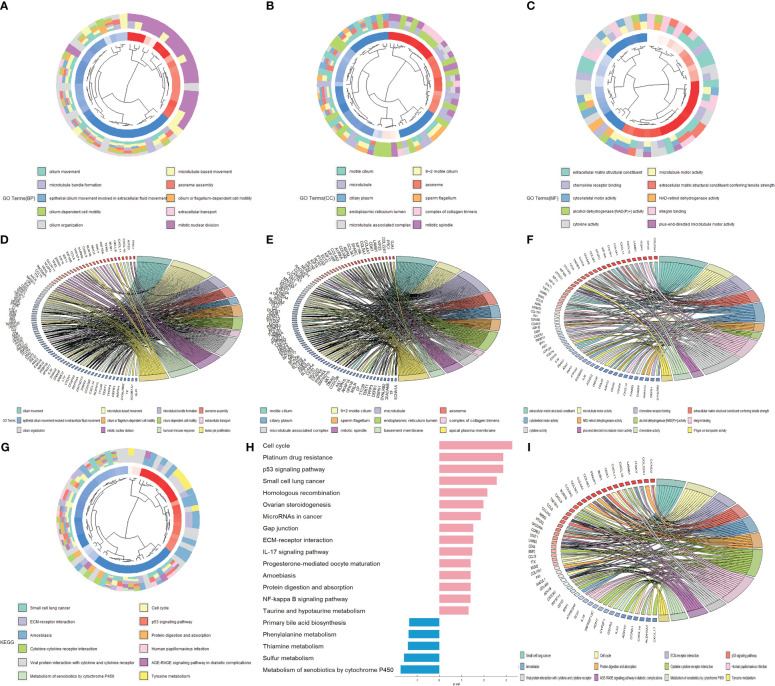
Enrichment analysis of the overlapping DEGs. GO cluster plots show the Gene Ontology (GO) analysis of differentially expressed genes (DEGs) in three GO terms: biological process (BP) **(A)**, cellular component (CC) **(B)**, and molecular function (MF) **(C)**. The key genes are displayed in the top twelve pathways of each chord diagram: BP **(D)**, CC **(E)**, MF **(F)** and KEGG **(I)**. Kyoto Encyclopedia of Genes and Genomes (KEGG) pathway analysis for the overlapping DEGs **(G, H)**.

To further study the function of DEGs (appearing in at least four groups), we carried out enrichment analysis by GOplot and the clusterProfiler R package. The GO analysis showed that the following BPs (biological processes) were notably enriched among the DEGs: axoneme assembly, cilium movement and microtubule-based movement. The CCs (cellular components) were mainly enriched in motile cilia, microtubules, axonemes and mitotic spindles, and MFs (molecular functions), such as extracellular matrix structural constituent and microtubule motor activity, were highly associated with the DEGs (**Figures 3A–F**). The KEGG pathway analysis revealed that DEGs in NPC were mainly enriched in the cell cycle, platinum drug resistance, p53 signaling pathway, metabolism of xenobiotics by cytochrome P450, amoebiasis and viral protein interaction with cytokine receptor (**Figures 3G, H**). Among all three GO terms and KEGG pathway analyses, we classified primary pathway-relevant DEGs through a chordal graph. These pathways included These pathways included BP (BUB1, CXCL10, SLPI, GNLY, PRC1, TPPP3) (**Figure 3D**); CC(CDK1, GJA1, COL22A1, LAMB1) (**Figure 3E**); MF (DYNLRB2, DEFB1, KIF23) (**Figure 3F**); and KEGG(CDK1, CXCL17, CYP2F1) (**Figure 3I**), which contributed to critical directions for our future studies. These enrichment pathways indicated that the DEGs were involved in tumor migration, tumor microenvironment and pathogenesis of NPC.

### Multiple network analysis of DEGs and identification of DEG mutation spectrum

**Figure 4:**
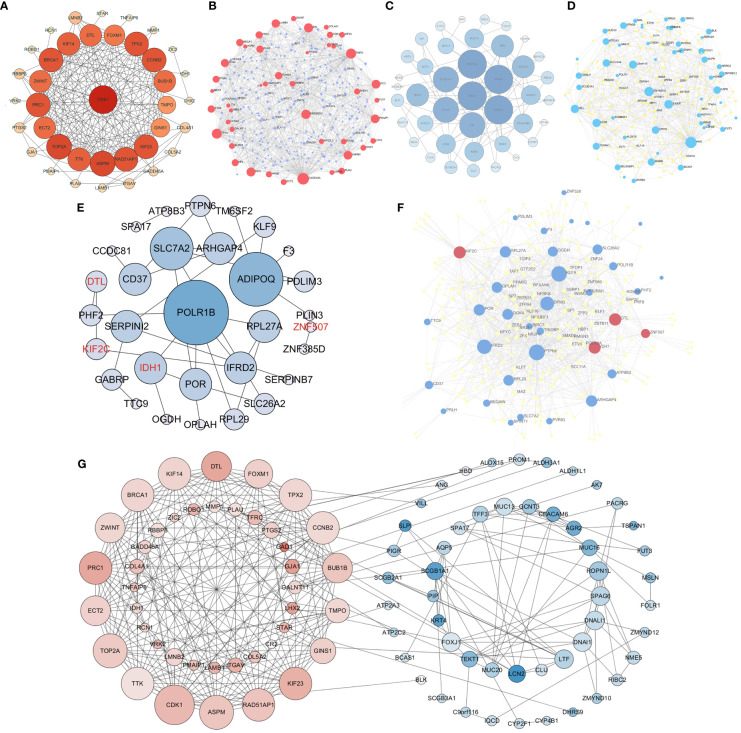
Protein−protein interaction (PPI) network for differentially expressed genes and construction of a transcription factor (TF)-gene network. PPI network for upregulated DEGs **(A)**. TF-gene interaction network of upregulated DEGs **(B)**. PPI network for downregulated DEGs **(C)**. TF-gene interaction network of downregulated DEGs **(D)**. PPI network and TF-gene interaction network of DEGs from the survival model **(E, F)**. The interaction between upregulated DEGs and downregulated DEGs **(G)**. Red nodes are upregulated DEGs, blue nodes are downregulated DEGs, and purple/yellow nodes represent transcription factors (TFs). The red font represents upregulated DEGs from survival models.

To further explore the relationship among these overlapping DEGs at the protein level, we constructed a protein−protein interaction (PPI) network. Based on the network analysis of Cytoscape, 16 upregulated DEGs, CDK1, DTL, FOXM1, TPX2, CCNB2, BUB1B, KIF23, RAD51AP1, ASPM, TTK, TOP2A, ECT2, PRC1, ZWINT, BRCA1 and IDH1, were identified as hub genes with degrees>15 (**Figure 4A**). Nine downregulated DEGs, SCGB1A1, ROPN1L, FOXJ1, LTF, DNALI1, TFF3, SPAG6, AGR2, and CEACAM6, were confirmed as hub genes with degrees>5 (**Figure 4C**). Moreover, the TF-gene interaction network showed that ELF1, SMAD5, TFDP1 and MAZ were highly related to DEGs, which played key roles in DEG transcription (**Figures 4B, D**). Furthermore, POLR1B and PTPN6 play a key role as nodes in the network diagram drawn by DEGs (**Figures 4E, F**). When all the DEGs were analyzed together, the results showed that the upregulated and downregulated hub DEGs were related to each other (CDK1-BLK, MMP1-LCN2, KIF14-VILL) (**Figure 4G**). In summary, these results indicated that interactions between DEGs could contribute to the occurrence and development of NPC.

Moreover, we used the cBioProtal Web tools online to carry out genetic mutation detection based on NPC datasets. Accordingly, 32 DEGs were found in the top mutation, including 12 upregulated DEGs (**Supplementary Figure S1A**) and 20 downregulated DEGs (**Supplementary Figure S1B**). Among them, TTN, FAT2, KLHDC7A and TNXB were highly associated with survival prognosis (**Supplementary Figure S2**). This result indicated that a considerable number of valuable DEGs were mutated in NPC and were associated with the survival of NPC.

### Survival analysis of the overlapping DEGs

**Figure 5:**
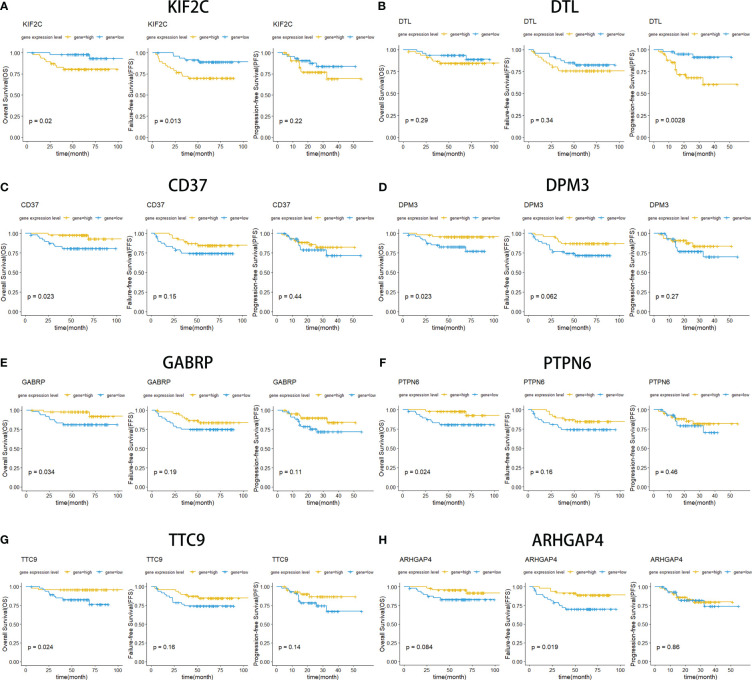
Kaplan−Meier curves for overall survival (OS), failure-free survival (FFS) and progression-free survival (PFS). OS survival curves comparing patients with high (yellow) and low (blue) gene expression in nasopharyngeal carcinoma, including KIF2C **(A)**, DTL **(B)**, CD37 **(C)**, DPM3 **(D)**, GABRP **(E)**, PTPN6 **(F)**, TTC9 **(G)**, and ARHGAP4 **(F)**.

Kaplan−Meier survival curves were drawn for NPC patients based on their overall survival, progression-free survival and failure-free survival information. Eight overlapping DEGs were screened out from more than 1000 overlapping DEGs, which have a strong relationship with patient survival (**Figure 5**). Among them, two upregulated DEGs (KIF2C and DTL) contributed to poor prognosis with lower survival rates (**Figures 5A, B**). Meanwhile, six downregulated DEGs (CD37, DPM3, GABRP, PTPN6, TTC9, ARHGAP4) were also screened out with a strong association with decreased OS, FFS and PFS (**Figures 5C–H**). These findings validated that these identified DEGs were closely related to the prognosis of the patients.

### Multivariate Cox regression analysis of DEGs and construction of the survival prediction model

**Figure 6:**
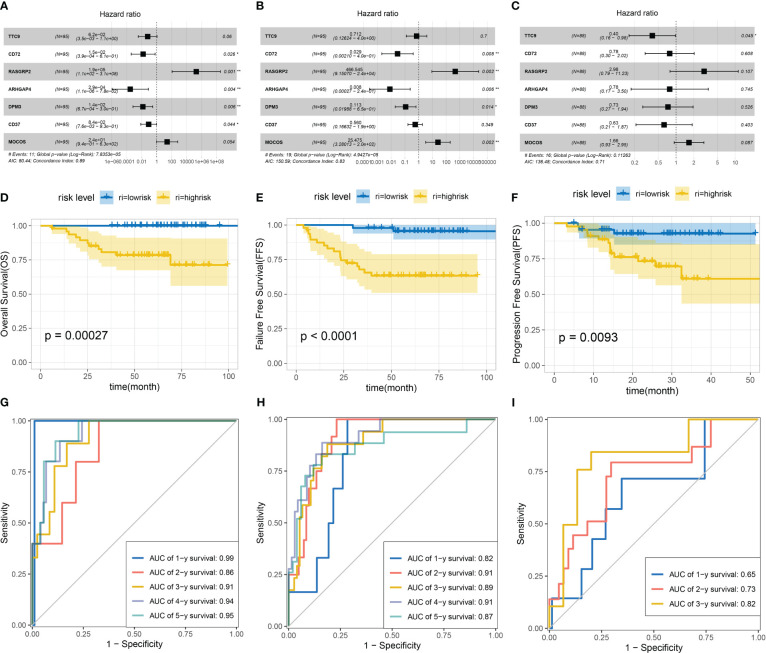
Construction of multivariate Cox regression analysis and survival models based on overall survival (OS), failure-free survival (FFS) and progression-free survival (PFS). Multivariate Cox forest figures were based on OS **(A)**, FFS **(B)** and PFS **(C)**. Survival models were constructed based on OS **(D)**, FFS **(E)** and PFS **(F)**. ROC curves for three models: OS **(G)**, FFS **(H)** and PFS **(I)**.

After selected genes were subjected to multiple Cox analysis, the DEG risk forest diagrams were constructed. According to the calculation of the risk score, patients were divided into high-risk and low-risk groups (**Supplementary Data Sheet 4**). Remarkably, some of the selected genes were present in the overlapping DEGs (identified from the five groups) (**Supplementary Data Sheets 1**, **2**). In-depth analysis revealed that seven DEGs (MOCOS, RASGRP2, CD37, DPM3, CD72, TTC9, and ARHGAP4) play a critical role in the survival of patients with NPC (**Figures 6A–C**). Notably, the DEGs we selected can jointly construct three survival prediction models (OS: p value=0.00044; FFS: p value<0.0001; PFS: p value<0.0001). The survival prognosis of patients classified into the high-risk group in these three models was significantly worse than that of patients in the low-risk group (**Figures 6D–F**). The value of the ROC curve is close to one, which proves that the reliability of the three models is very high (**Figures 6G–I**). Combined with the above PPI network analysis, several DEGs as the nodes in the network diagram were the key genes in the survival model, including POLR1B and PTPN6. Together, these models could be applied to predict the prognosis of patients, which indicated that the selected DEGs were positive factors affecting the prognosis of the patients.

### The significance of clinical information for genetic screening

Correlation analysis was performed between DEGs and clinical information (TNM staging system), and six overlapping DEGs were closely related to clinical information. The upregulated DEGs (TOP2A, INSM1) increased with the progression of T and N staging (**Supplementary Figures S3A, B**). The downregulated DEGs (GABRP, DPM3, CD37 and ARHGAP4) decreased with the progression of T and N staging (**Supplementary Figures S3C–F**). This result suggested that the more malignant the cancer was, the higher the upregulated genes and the lower the downregulated genes were expressed. Simultaneously, we assessed the clinical information about staging and found that the upregulated DEG TOP2A expression in stages III/IV was higher than that in stages I/II (**Supplementary Figure S3A**), which might be critical evidence of tumor aggressiveness (43). Additionally, there was obviously decreased CD37 expression with increasing T stage (**Supplementary Figure S3E**). Moreover, we detected a significant reduction in LCK in the N3 group, which might mean that low LCK expression is related to regional lymph node metastasis (**Supplementary Figure S4**). It is also worth mentioning that S100P, CEACAM6, MSLN and KRT23 also showed a high correlation with the progress of T and N staging (**Supplementary Figure S4**). Together, these results indicated that the expression levels of the screened genes were strongly associated with tumor progression.

### Verification of the expression level of the above screened DEGs through qRT−PCR, WB, IHC and single cell sequencing data(scRNA-seq)

**Figure 7:**
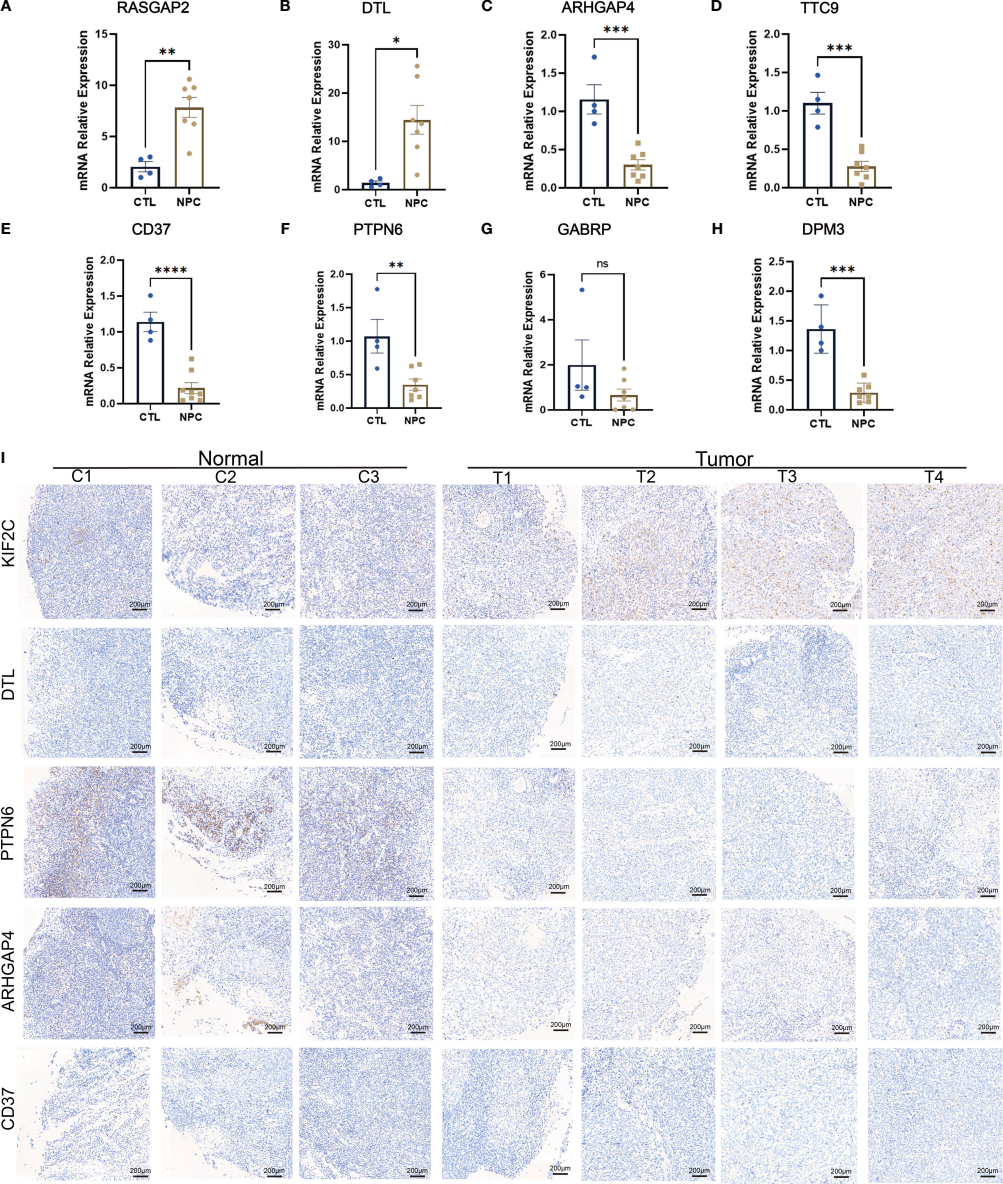
PCR results of several identified differentially expressed genes (DEGs), including KIF2C **(A)**, DTL **(B)**, ARHGAP4 **(C)**, TTC9 **(D)**, CD37 **(E)**, PTPN6 **(F)**, GABRP **(G)**, and DPM3 **(H)**. Immunohistochemistry results of KIF2C, DTL, PTPN6, ARHGAP4 and CD37 **(I)**. ns: P>0.05; *: P<0.05; **: P<0.01; ***: P<0.001; ****: P<0.0001.

To further validate the differential expression of the key genes above, we investigated the expression levels of RASGRP2, ARHGAP4, CD37, DPM3, GAPRB, TTC9, PTPN6 and DTL in NPC specimens and control specimens. Each of these genes is associated with a poor prognosis in patients with NPC, and they are all overlapping DEGs (**Supplementary Data Sheets 1**, **2**). At the same time, CD37, DTL, and ARHGAP4 are also hub genes. Two genes (RASGRP2 and DTL) (**Figures 7A, B**, **Supplementary Figure S5A**) were upregulated, and five genes (ARHGAP4, TTC9, CD37, PTPN6, GABRP and DPM3) (**Figures 7C–H**, **Supplementary Figure S5A**) were downregulated in NPC, which is consistent with our previous results. Furthermore, we were able to identify a few genes that displayed significant differences in expression based on the WB results, including KIF2C, DTL, CD37, ARHGAP4, and PTPN6. DTL, KIF2C, ARHGAP4, PTPN6, and CD37 were differentially expressed, aligning with our computational and experimental findings (**Supplementary Figures S6A–G**). Additionally, we found that 10 up-regulated DEGs and 11 down-regulated DEGs (**Table 2**), grouped into five categories each, corresponded with expression levels in scRNA-seq data; LTF was the exception, showing no significant difference (**Supplementary Figures S6H–K**). To validate these findings, immunohistochemical staining was performed, and the results showed that KIF2C and DTL exhibited positive staining in tumor samples, which aligns with our above obtained results. Conversely, PTPN6, ARHGAP4, and CD37 demonstrated weaker positive staining in tumor samples, consistent with our previous findings (**Figure 7I**). The IHC results also illustrated that up-regulated KIF2C was associated with patients’ poor prognosis (**Supplementary Figures S5B–D**). Together, these results implied that the expression levels of key genes screened from mega-data bioinformatics analysis basically reflected the real situation in patients with NPC, which could predict the prognosis of NPC patients.

## Discussion

In our study, an extensive dataset sourced from reputable databases was meticulously analyzed through bioinformatics methods to screen for differentially expressed genes (DEGs) in nasopharyngeal carcinoma (NPC). This approach allowed us to construct a unique survival model for NPC patients, opening up exciting new avenues for research. A flow chart summarizing the overall results was shown in **Supplementary Figure S7**. Currently, there is no overall survival information for NPC in public databases, such as GEO and TCGA, so it has not been reported previously that using mega-data to identify the DEGs, carrying out in-depth bioinformatics analysis and then combining clinical information to analyze the survival of NPC patients. First, we selected 15 datasets from GEO and merged them into five groups, including 46 controls and 160 NPC patients, with increasing accuracy and credibility. On this basis, further bioinformatics analysis identified 2142 upregulated DEGs and 3857 downregulated DEGs. Significantly, DPM3 was differentially expressed in European populations, while CD37, ARHGAP4, and CD72 showed differential expression in Asian cohorts. Furthermore, survival analysis was performed, and survival models were constructed based on these identified DEGs, with the minority of them being reported in a previous study of NPC (44). Finally, the consistent differential expression of these DEGs in NPC samples was confirmed by qRT−PCR, WB and IHC, which confirmed the accuracy of mega-data bioinformatics analysis. All of the above findings from mega-data bioinformatics analysis provide insights into the novel prognostic biomarkers of NPC, which suggests that they may serve special targets in the treatment of NPC.

Our results are derived from the analysis of mega-data, endowing them with enhanced reliability and accuracy in contrast to studies conducted with limited sample sizes (45, 46). First, several DEGs from our screening were consistent with previous reports (11, 47). Hub genes identified by previous experiments, such as CD37 and PTPN6, were also discovered in our study (48, 49). In addition, hub genes that were used to construct models also appeared in previous studies. ARHGAP4 plays a pivotal role in governing the intricate processes of cell migration and invasion in pancreatic cancer through modulation of the HDAC2/β-catenin signaling pathway (50). CD37 emerges as an autonomous prognostic determinant, exhibiting immunosuppressive properties within the domain of diffuse gliomas (51). Furthermore, DPM3/prostin-1, a novel gene regulated by phospholipase C-gamma, displays a correlation with prostate tumor invasion and represents a noteworthy target for inhibiting invasive behaviors (52). Moreover, the consistent differential expression of these DEGs between the profile screen and verification of tissues was confirmed by qRT−PCR, WB and IHC. These results indicate the accuracy of the mega-data bioinformatics analysis.

In our research, more sample clinical information was collected, especially survival information, which is distinct from other studies (53, 54). Previous studies often performed survival analysis by using GSE102349 reported in 2017, which only contained progression-free survival (PFS) information for NPC patients (55–57). The survival analysis results they obtained were limited to a small number of samples, leading to significant biases in the outcomes. Moreover, owing to the absence of clinical information on NPC, some studies resorted to using data from head and neck squamous cell carcinoma (HNSCC) for survival analysis. However, since the HNSCC database hardly includes any information on NPC, which is a tumor type vastly distinct from HNSCC, the derived survival results are virtually devoid of any meaningful reference value (8–11). To overcome these limitations, we obtained NPC RNA expression profiles from GSE132112 and corresponding clinical information from Professor Jun Ma at Sun Yat-sen University. This allowed us to establish a comprehensive survival analysis by exploring the relationship between DEGs and overall survival (OS), failure-free survival (FFS), and progression-free survival (PFS), uniquely constructing survival models using NPC-specific DEGs. This allows for our subsequent analysis of clinical information and survival analysis to better reflect the real situation. Therefore, with the support of these crucial clinical data, our analysis is more capable of reflecting the reality accurately.

In our study, we must consider the inherent biases associated with bioinformatics analyses, including the potential for skewed data representation and the complexity of merging datasets from diverse sources, which may be inaccurate. Moreover, the generalizability of our prognostic model across different ethnicities and regions remains uncertain, given the variable prevalence and genetic landscape of NPC globally. Lastly, while qRT-PCR and IHC have validated the expression of certain DEGs, the absence of functional assays in our study leaves the direct impact of these genes on NPC progression and patient outcomes unverified.

To advance our understanding of the functional roles that hub genes and DEGs play in NPC, future research should prioritize targeted *in vitro* and *in vivo* studies, such as gene knockdown and overexpression experiments, to examine their effects on tumor biology and treatment response. Prospective clinical trials are warranted to validate our prognostic model, ensuring its applicability and integration into clinical workflows for improved patient management. Furthermore, concerted efforts to develop novel targeted therapies based on our identified DEGs, particularly those unexplored in NPC context, and to investigate the tumor-immune interface, will be crucial for pioneering new avenues in NPC treatment and immunotherapeutic strategies.

In summary, our study confirmed the DEGs between NPC and normal tissue that are crucial for the prognosis of NPC through bioinformatics methods from mega-data. Furthermore, our results imply that mega-data analysis would provide more accurate predictions and hints in NPC prognosis. Overall, our study not only integrated mega-data analysis and clinical information but also constructed a promising survival model specific to NPC patients by combining several key genes as novel prognostic biomarkers, which suggests that they may serve special targets in the treatment of NPC.

## Data availability statement

Publicly available datasets were analyzed in this study. This data can be found here: https://www.ncbi.nlm.nih.gov/geo/, GSE12452,GSE64634, GSE15047, GSE15903, GSE36972, GSE48501, GSE100193, GSE79571, GSE119020, GSE127848, GSE128502, GSE13597, GSE53819, GSE40290, GSE34573, GSE102349, and GSE132112 and https://explore.data.humancellatlas.org/projects/111d272b-c25a-49ac-9b25-e062b70d66e0.

## Ethics statement

The studies involving humans were approved by West China Hospital of Sichuan University. The studies were conducted in accordance with the local legislation and institutional requirements. The participants provided their written informed consent to participate in this study.

## Author contributions

YT: Data curation, Formal analysis, Funding acquisition, Investigation, Methodology, Software, Visualization, Writing – original draft. JiaoZ: Formal analysis, Funding acquisition, Investigation, Supervision, Validation, Writing – review & editing. KL: Formal analysis, Investigation, Methodology, Writing – original draft. RL: Formal analysis, Methodology, Validation, Writing – original draft. JingZ: Formal analysis, Investigation, Methodology, Supervision, Visualization, Writing – original draft. ZW: Methodology, Supervision, Validation, Writing – original draft. LL: Data curation, Resources, Supervision, Writing – original draft. JZ: Writing – original draft. XF: Writing – original draft. BD: Writing – original draft. JD: Conceptualization, Funding acquisition, Supervision, Writing – review & editing.

## Supplementary material

Supplementary Figure 1 Mutation plots of differentially expressed genes (DEGs). The top 12 most mutated upregulated DEGs **(A)** and top 20 most mutated downregulated DEGs **(B)** in NPC.

Supplementary Figure 2 Kaplan−Meier curves for overall survival (OS), failure-free survival (FFS) and progression-free survival (PFS). OS survival curves comparing patients with high (yellow) and low (blue) gene expression in nasopharyngeal carcinoma, including TTN **(A)**, FAT2 **(B)**, KLHDC7A **(C)**, TNXB **(D)**, MOCOS **(E)**, and CD72 **(F)**.

Supplementary Figure 3 Relationship between gene expression and different clinical information. Boxplot of expression differences of DEGs grouped by staging of cancer and tumor-node-metastasis (TNM) staging system, including TOP2A **(A)**, INSM1 **(B)**, GABRP **(C)**, DPM3 **(D)**, CD37 **(E)**, and ARHGAP4 **(F)**.

Supplementary Figure 4 Relationship between gene expression and different clinical information. Boxplot of expression differences of DEGs grouped by staging of cancer and tumor-node-metastasis (TNM) staging system, including LCK **(A)**, MSLN **(B)**, S100P **(C)**, KRT23 **(D)** and CEACAM6 **(E)**.

Supplementary Figure 5 Western blotting (WB) and survival analysis based on IHC results. **(A)** WB results of KIF2C, DTL, ARHGAP4, CD37 and PTPN6. **(B–D)** Survival analysis based on IHC results for KIF2C, PTPN6 and ARHGAP4.

Supplementary Figure 6 Validation differentially expressed genes using scRNA-seq data. **(A)** Umap plot for nasopharyngeal carcinoma samples and control samples. Blue: nasopharyngeal carcinoma. Red: Control. **(B–F)** Umap plots for the expression levels of DTL, KIF2C, ARHGAP4, PTPN6 and CD37.

Supplementary Figure 7 A flow chart of the overall results.

Supplementary Data Sheet 1 Upregulated differentially expressed genes (DEGs).

Supplementary Data Sheet 2 Downregulated differentially expressed genes (DEGs).

Supplementary Data Sheet 3 Upregulated and Downregulated differentially expressed genes with |log2FC|>1.5 and p<0.05 (DEGs).

Supplementary Data Sheet 4 Cox regression analysis of differentially expressed genes (DEGs).
