# Supplementary figures and images for: Novel prognostic biomarkers in nasopharyngeal carcinoma unveiled by mega-data bioinformatics analysis
Source: Front Oncol. 2024 May 24;14:1354940. doi: 10.3389/fonc.2024.1354940 (PMC11157084; doi:10.3389/fonc.2024.1354940)

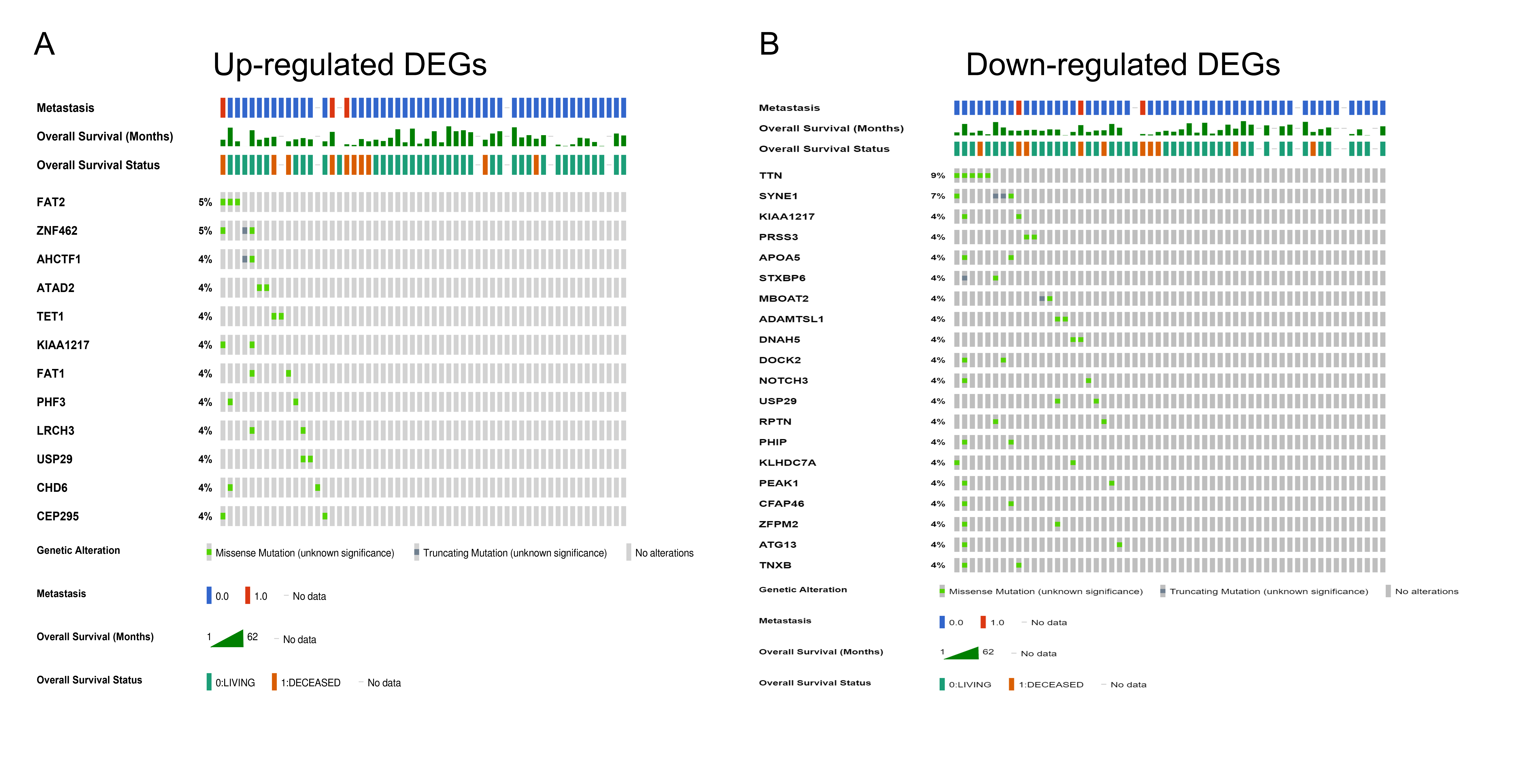

Supplement: Supplementary Figure 1 — Mutation plots of differentially expressed genes (DEGs). The top 12 most mutated upregulated DEGs (A) and top 20 most mutated downregulated DEGs (B) in NPC. [file Image_1.jpeg]

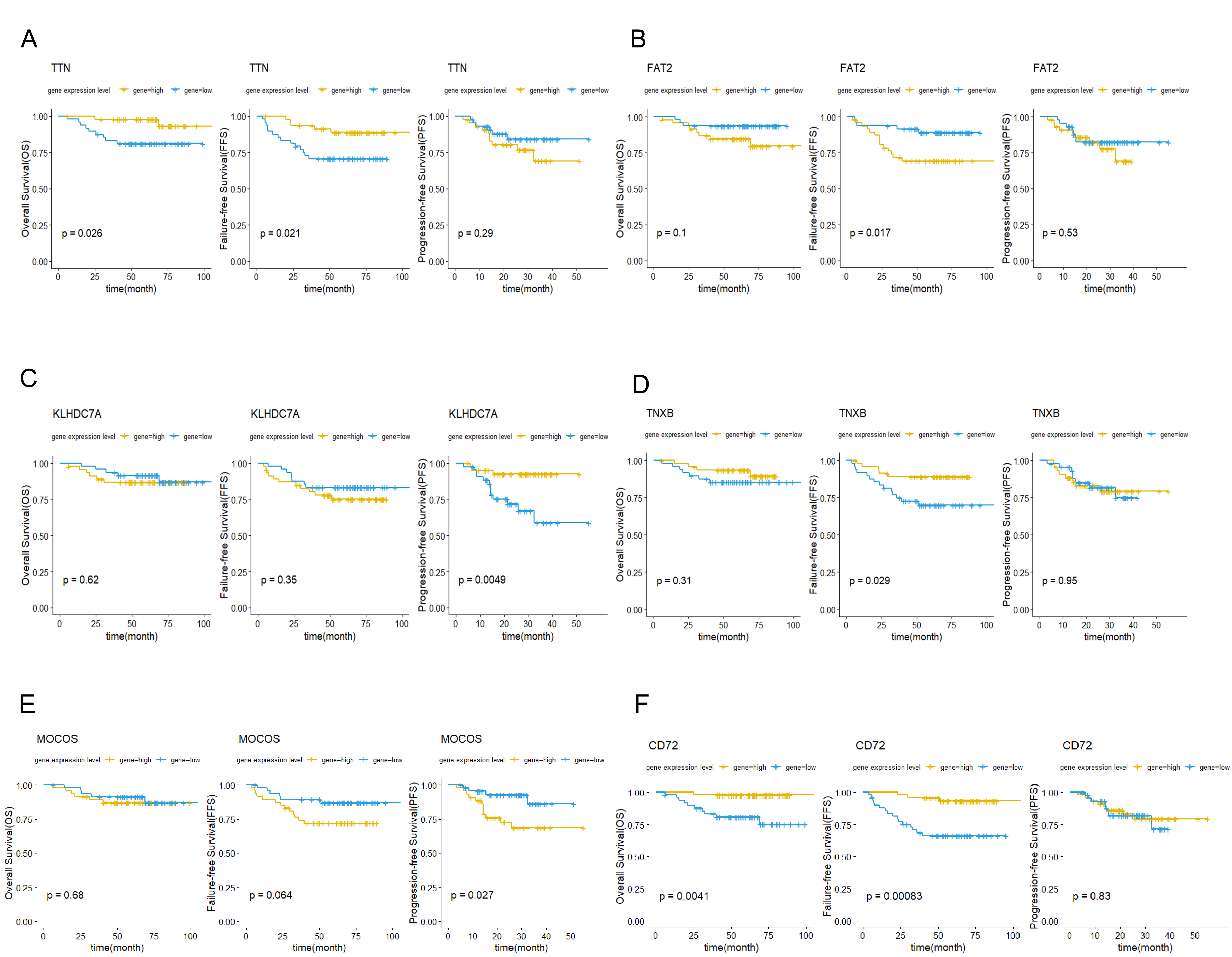

Supplement: Supplementary Figure 2 — Kaplan−Meier curves for overall survival (OS), failure-free survival (FFS) and progression-free survival (PFS). OS survival curves comparing patients with high (yellow) and low (blue) gene expression in nasopharyngeal carcinoma, including TTN (A), FAT2 (B), KLHDC7A (C), TNXB (D), MOCOS (E), and CD72 (F). [file Image_2.jpeg]

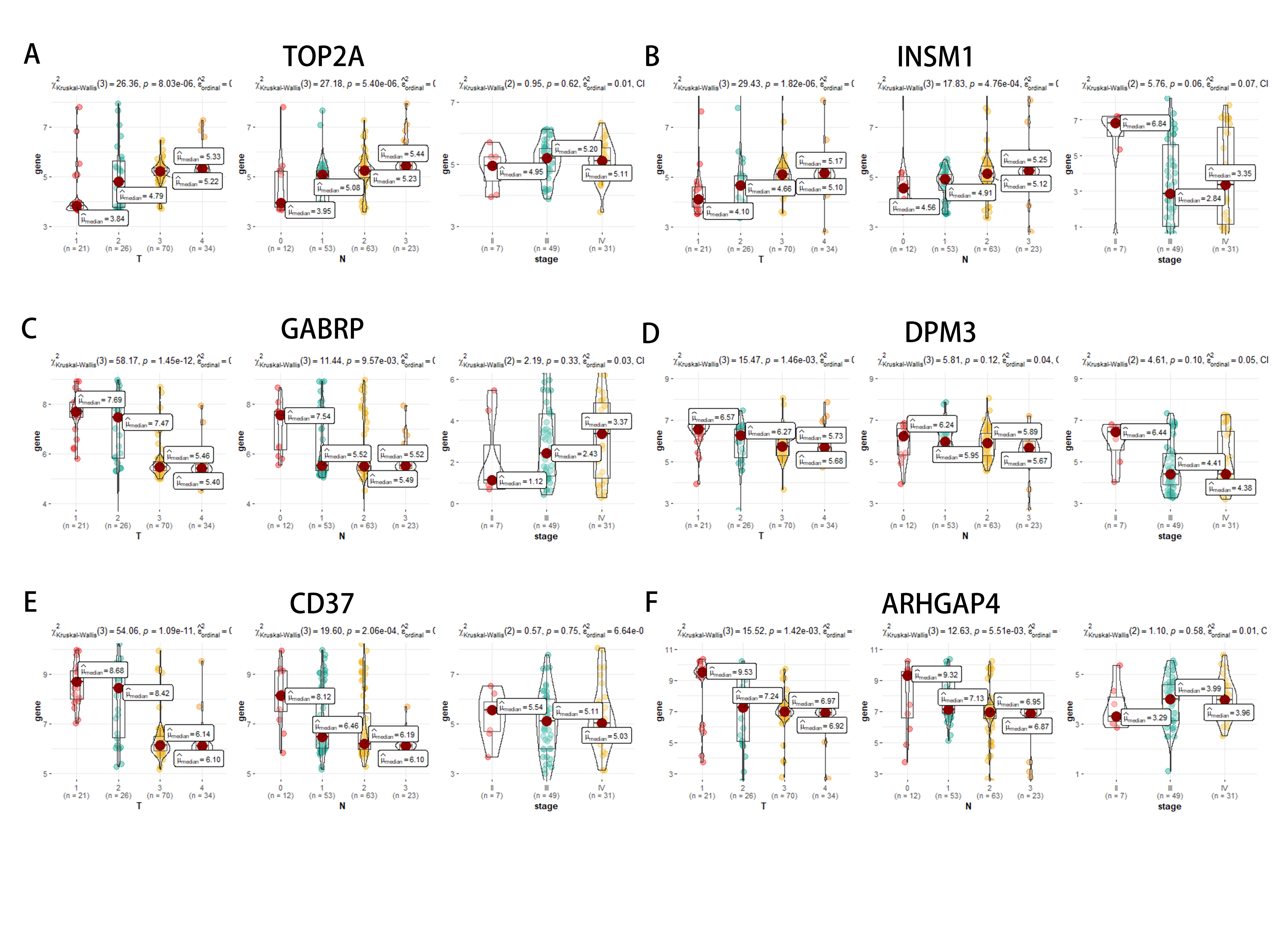

Supplement: Supplementary Figure 3 — Relationship between gene expression and different clinical information. Boxplot of expression differences of DEGs grouped by staging of cancer and tumor-node-metastasis (TNM) staging system, including TOP2A (A), INSM1 (B), GABRP (C), DPM3 (D), CD37 (E), and ARHGAP4 (F). [file Image_3.jpeg]

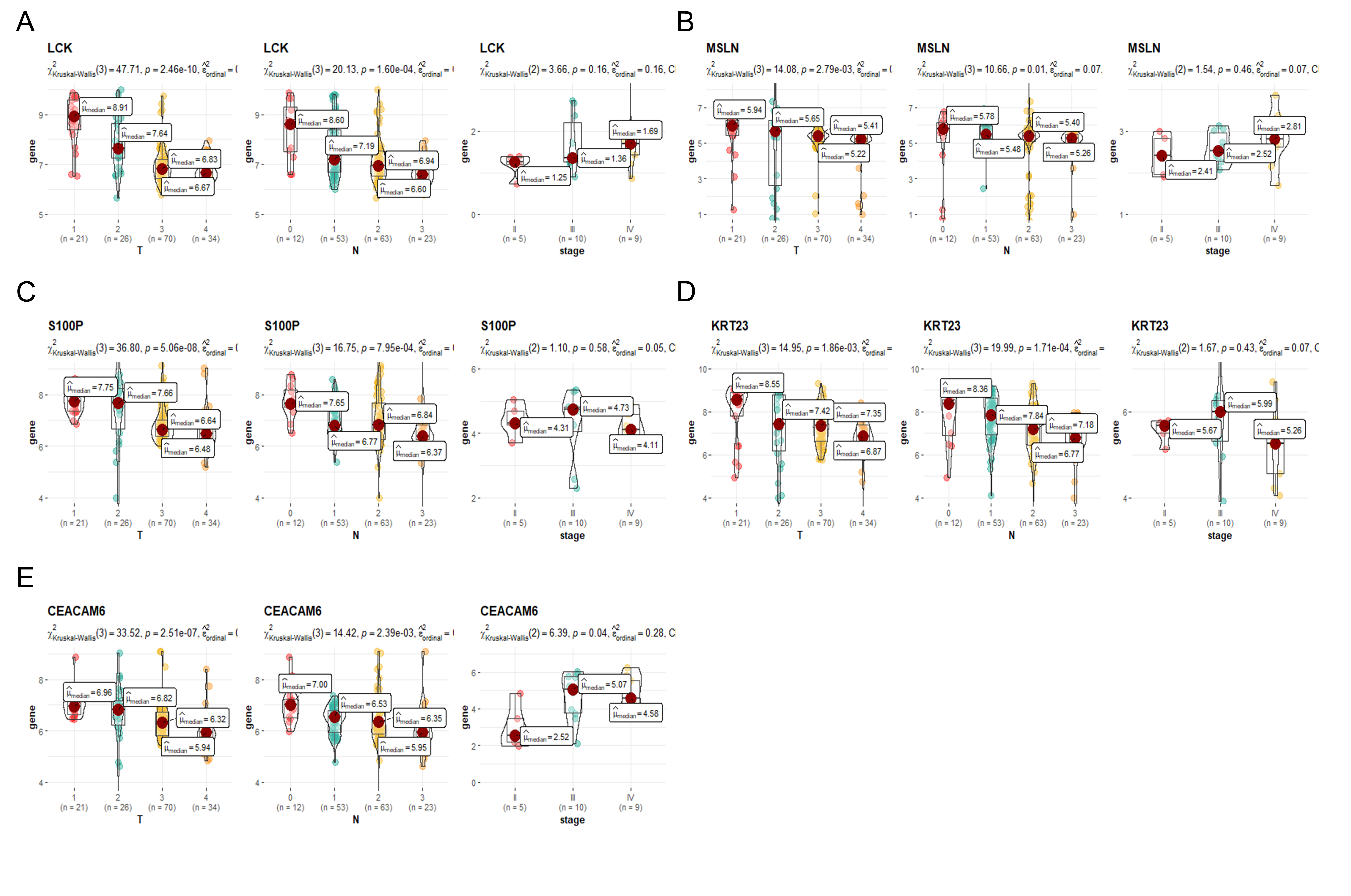

Supplement: Supplementary Figure 4 — Relationship between gene expression and different clinical information. Boxplot of expression differences of DEGs grouped by staging of cancer and tumor-node-metastasis (TNM) staging system, including LCK (A), MSLN (B), S100P (C), KRT23 (D) and CEACAM6 (E). [file Image_4.jpeg]

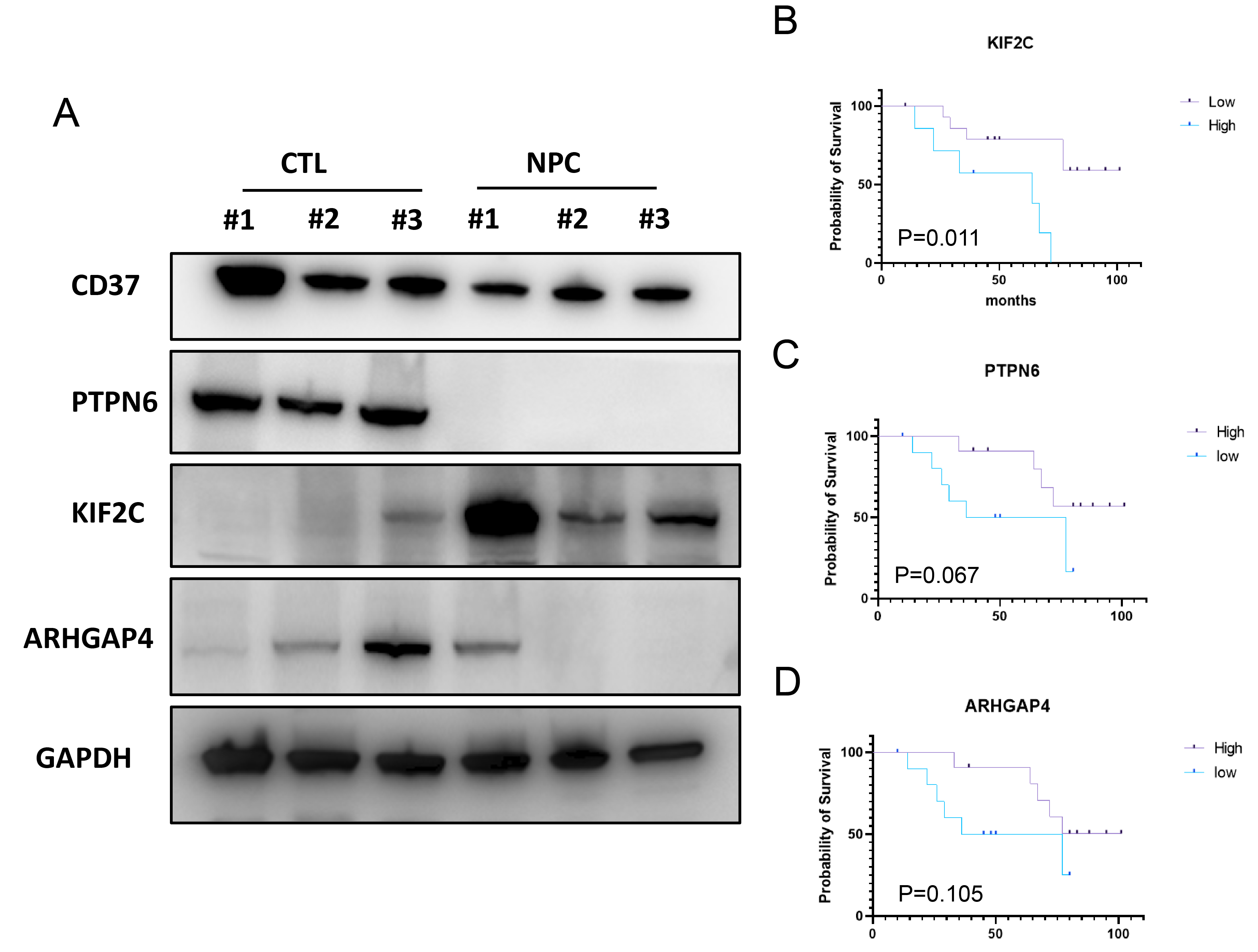

Supplement: Supplementary Figure 5 — Western blotting (WB) and survival analysis based on IHC results. (A) WB results of KIF2C, DTL, ARHGAP4, CD37 and PTPN6. (B–D) Survival analysis based on IHC results for KIF2C, PTPN6 and ARHGAP4. [file Image_5.jpeg]

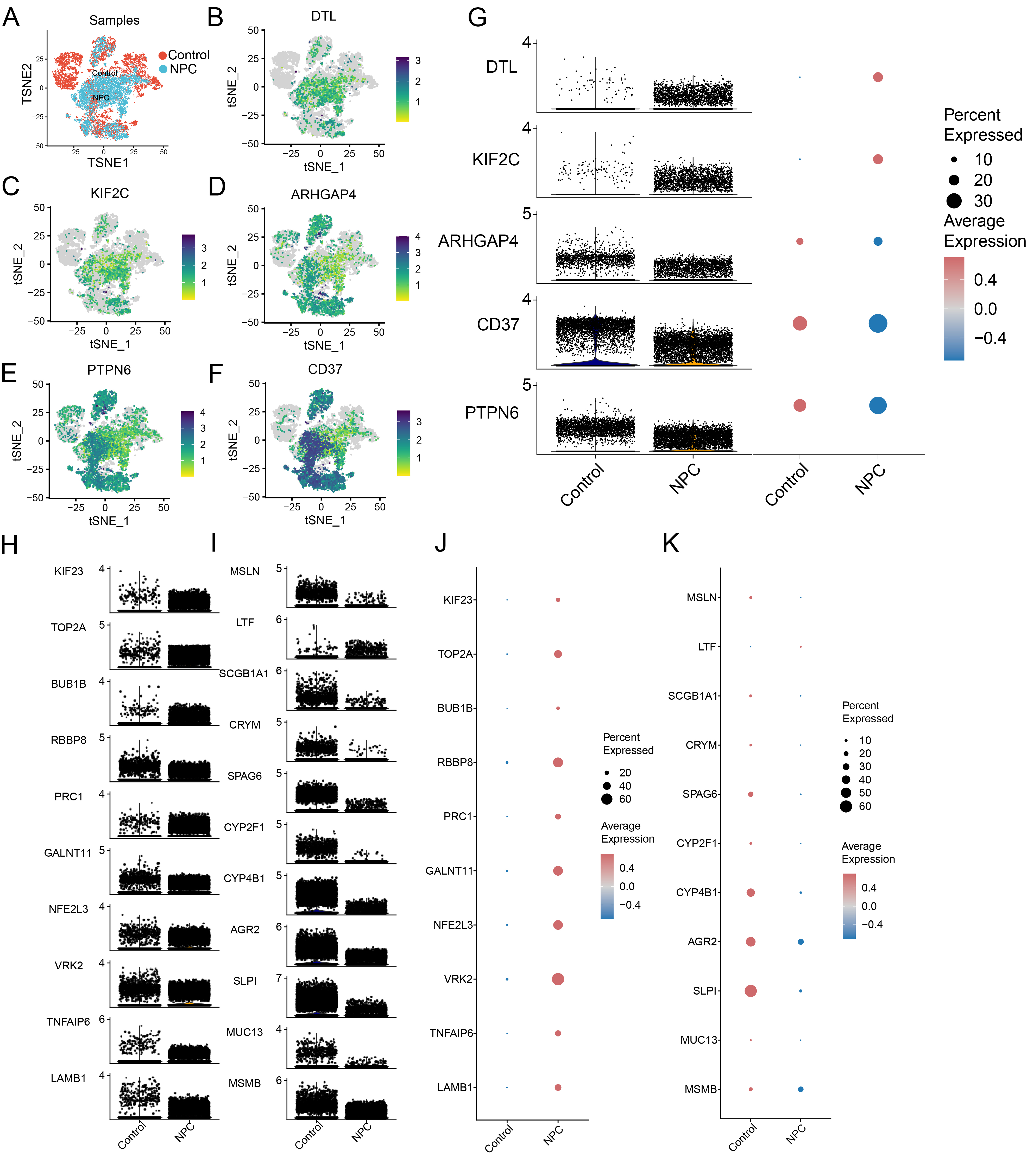

Supplement: Supplementary Figure 6 — Validation differentially expressed genes using scRNA-seq data. (A) Umap plot for nasopharyngeal carcinoma samples and control samples. Blue: nasopharyngeal carcinoma. Red: Control. (B–F) Umap plots for the expression levels of DTL, KIF2C, ARHGAP4, PTPN6 and CD37. [file Image_6.jpeg]

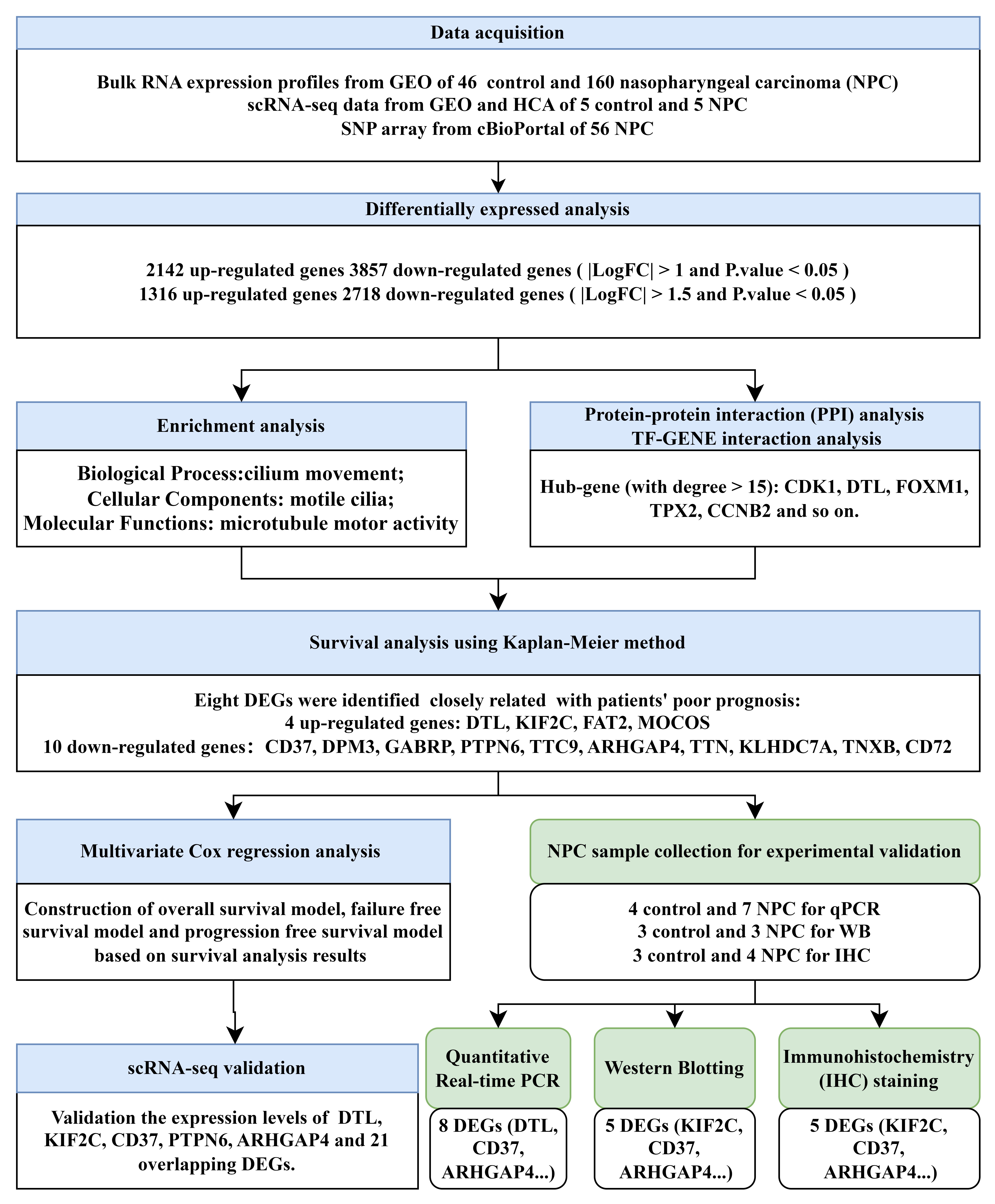

Supplement: Supplementary Figure 7 — A flow chart of the overall results. [file Image_7.jpeg]
