# Supplementary material for: Novel prognostic biomarkers in nasopharyngeal carcinoma unveiled by mega-data bioinformatics analysis
Source: Front Oncol. 2024 May 24;14:1354940. doi: 10.3389/fonc.2024.1354940 (PMC11157084; doi:10.3389/fonc.2024.1354940)
Supplement: Supplementary file 12 [file Table_1.docx]

Table S1, Descriptive statistics of immunohistochemical variables.

| Variables | Low-expressed | High-expressed | Positive rate |
| --- | --- | --- | --- |
| KIF2C | 15 | 7 | 13.43% |
| PTPN6 | 11 | 11 | 9.16% |
| ARHGAP4 | 11 | 11 | 9.89% |
